# Controlling the Molecular Weight of Lignosulfonates by an Alkaline Oxidative Treatment at Moderate Temperatures and Atmospheric Pressure: A Size-Exclusion and Reverse-Phase Chromatography Study

**DOI:** 10.3390/ijms18122520

**Published:** 2017-11-24

**Authors:** Chamseddine Guizani, Dominique Lachenal

**Affiliations:** French National Centre for Scientific Research (CNRS), University Grenoble Alpes, Grenoble INP, LGP2, F-38000 Grenoble, France; dominique.lachenal@pagora.grenoble-inp.fr

**Keywords:** lignosulfonates, depolymerisation, size-exclusion chromatography

## Abstract

The molecular weights of lignosulfonates (LSs) are modified by a rather simple process involving an alkaline oxidative treatment at moderate temperatures (70–90 °C) and atmospheric pressure. Starting from LSs with an average molecular weight of 90,000 Da, and using such a treatment, one can prepare controlled molecular weight LSs in the range of 30,000 to 3500 Da based on the average mass molecular weight. The LS depolymerisation was monitored via reverse-phase and size-exclusion chromatography. It has been shown that the combination of O_2_, H_2_O_2_ and Cu as a catalyst in alkaline conditions at 80 °C induces a high LS depolymerisation. The depolymerisation was systemically accompanied by a vanillin production, the yields of which reached 1.4 wt % (weight percentage on LS raw basis) in such conditions. Also, the average molecular weight and vanillin concentration were correlated and depended linearly on the temperature and reaction duration.

## 1. Introduction

Depletion of natural and fossil resources, global warming, soil loss and degradation of agricultural lands are all symptoms of a world in degeneration and mankind “overconsuming the nature” [1]. Regarding these alarming signs, a shift of paradigm seems necessary for a safe and sustainable future for humans and nature [2].

Biomass constitutes the largest source of renewable carbon in the world and thus can be viewed as a pillar in building this sustainable future. Second-generation biomass, such as woody biomass which is available in huge quantities in the world, can be the starting material for the production of biofuels and bio-based materials, thus ensuring, at least in part, the shift from an oil-based to a bio-based economy, which constitutes a real opportunity and leverage for a more sustainable future [3].

Pulp and paper industries can be already viewed as bio-refineries, as they transform the woody biomass into value-added products (pulp, paper, lignosulfonates, etc.) and fuels (hemicelluloses and lignin in the cooking liquor which are concentrated and burnt afterwards for energy recovery). Nevertheless, the full potential of wood valorization in such industries is clearly not reached. Hemicelluloses and lignin can be more efficiently used for other value-added applications rather than burnt. For instance, only 1–2% of the 50–70 million tons of lignin produced annually is used for the production of value-added products [4].

With the emergence of the biorefinery approach [5] and new paradigms concerning lignin, such as “lignin-first” [6], lignin does not appear anymore as the undesired byproduct of paper or second-generation bio-ethanol production processes, but a molecule with great potential to be used in many value-added applications such as for the production of vanillin or carbon fibers, or for the replacement of phenol compounds in wood adhesives that are widely used in wood products and furniture [7]. The old and well-rooted saying, “you can make almost everything out of lignin except money”, starts to break under the increasing force of innovative researchers all over the world.

The sulfite and Kraft processes (end of the 19th century) are the oldest and most well-known processes of wood pulping. The sulfite process has been largely replaced by the Kraft process since 1930 with the invention of the recovery boiler, which enabled the recovery and reuse of the inorganic pulping chemicals. Nevertheless, the sulfite process is maintained for the production of dissolving cellulose, with lignosulfonates (LSs) as byproduct. It shows also a renewed interest within the frame of the biorefinery approach.

Lignosulfonates are sulfonated lignins obtained during the sulfite cooking of wood. After a nucleophilic attack of HSO_3_^−^ on the carbons at the alpha and possibly gamma positions of the side chain of the lignin phenyl propane units (ppu), the lignin is solubilized and is recovered as LSs. LSs are also much less depolymerized than Kraft lignin due to much lower β-aryl ether bond cleavage.

LSs have many uses, such as animal feed, pesticides, surfactants, additives in oil drilling, vanillin precursors, stabilizers in colloidal suspensions, or as plasticizers in concrete admixtures. They account actually for 90% of the total market of commercial lignin, and the total annual worldwide production of lignosulfonates is approximately 1.8 million tons [8].

Sulfonated lignin-based products have been used as dispersants in cement admixtures and dye solutions more than in other applications, and their molecular weight and sulfonation degree are crucial in determining their efficiency [4,9]. The control of the molecular weight was also demonstrated to be highly relevant for the production of lignin-based carbon fibers with good mechanical properties [10] or for use as a dye dispersant [9]. More-recent applications dealing with 3-D printing of LSs and cellulose micro-fibrils [11] are also emerging and will probably require a control of LS molecular weight distribution (MWD). Controlling the molecular weight of LSs can be done by a separation technique, such as ultra-filtration (UF), to choose a defined molecular weight window for the lignin fragments [9]. In this sense, UF works well but is still time consuming.

The MWD can also be tailored by a controlled lignin depolymerisation. The depolymerisation of LS is usually studied in rather harsh conditions of temperature (>120 °C) and under oxygen partial pressure (>1 bar) with the aim to maximize monomers, especially vanillin [12,13,14]. Soft conditions in terms of temperatures and oxygen pressure are scarcely studied, probably because they do not match with the final aim of maximizing monomer production.

Other researchers were interested in the chemical modifications of LSs by alkaline hydrolysis at rather high temperatures of 116–180 °C for a subsequent use in phenolic resins to replace phenols [15]. The authors found a decrease of average mass molecular weight of LSs from 8512 to 4718 Da after treatment at 180 °C for 60 min, while the phenolic content increased from 1.15% to 1.72%, which conferred a higher reactivity of the LSs with formaldehyde. Also in [16], the authors reported a high decrease of methoxyl groups and sulfonate groups for H_2_O_2_-oxidized LS at a low temperature of 60 °C. The authors reported comparable mechanical properties for the resins, for which 30 wt % of phenol was replaced by the H_2_O_2_-oxidized LS.

A controlled LS molecular weight thus appears of high importance regarding its subsequent applications. The recovery of LSs can be done afterwards by a solvent evaporation or purification step [17].

For applications like adhesives and bioplastics, the lignin issued from the cellulose industry has too-high molecular weight and does not possess enough functional groups like free phenols and carboxyls. In the present work, we are interested in the possibility of converting LSs into lower-molecular-weight molecules (monomers and oligomers) which can be used for such applications.

Degradation of lignin by oxygen (possibly assisted by hydrogen peroxide) has been well documented. In their pioneering papers [18,19], J. Gierer and F. Imsgard clearly demonstrated the ability of these two oxidants to cleave some carbon–carbon linkages in the phenolic units of the lignin macromolecule. Later, A. Imai et al. [20] showed that both phenolic and nonphenolic moieties in Kraft lignin are extensively oxidized by oxygen in alkaline medium, and that the most plausible mechanism is the cleavage of alkyl–aryl ether linkages, a process that converts nonphenolic into phenolic moieties. Moreover, the formation of monomers (vanillin, syringaldehyde, etc.) in an oxygen delignification (bleaching) process has been demonstrated by Z. Wong et al. [21], confirming the interest of oxygen (and hydrogen peroxide, which acts in a very similar way) for lignin depolymerisation.

We propose in the present work a rather soft method for a controlled LS depolymerisation in an alkaline medium under an atmospheric pressure of oxygen (assisted or not by H_2_O_2_) and relatively low temperatures (70–90 °C). Following this method, we can reduce the LS molecular weight and make it more suitable (via the production of phenolic monomers and oligomers) for further applications in bioplastics, adhesives or carbon-fiber production. The LS depolymerisation was monitored by means of size-exclusion chromatography (SEC) and reverse-phase chromatography (RPC). Chromatographic methods and a homemade program were developed to monitor the reaction.

## 2. Results and Discussion

### 2.1. The Raw LS

**Figure 1:**
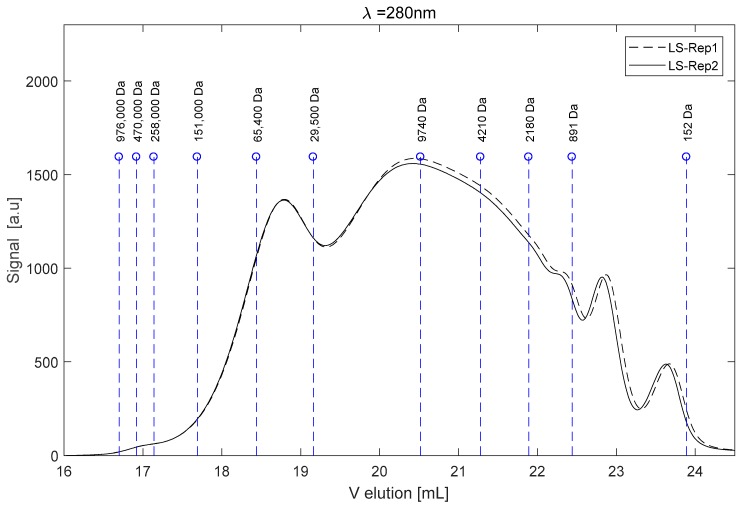
Eluogram of LS and peak elution volume of poly styrene sulfonate (PSS) calibrants.

Figure 1 shows an example of a SEC analysis of the LS sample in a 2 M NaOH solution. The elution volumes at peak positions for the different calibrants are shown by dashed stems. The measure was repeated twice and the analytical repeatability was quite good, as the relative difference between the two signals was less than 5% based on the average mass molecular weight ${\bar{M}}_{w}$.

The LS used for this work showed a high dispersity of the fragments’ molecular weights. The range was very wide, from 10^6^ Da to small molecules, the latter of which would be probably produced during the sulfite pulping. The calculated average mass and number molecular weights were respectively ${\bar{M}}_{w}$ = 90,357 Da and ${\bar{M}}_{n}$ = 16,172 Da. The polydispersity index (*PDI*) was about 5.6.

### 2.2. Monitoring the LS Depolymerisation

The LS depolymerisation in alkaline conditions at atmospheric pressure will be discussed based on the results of SEC and RP–HPLC.

**Figure 2:**
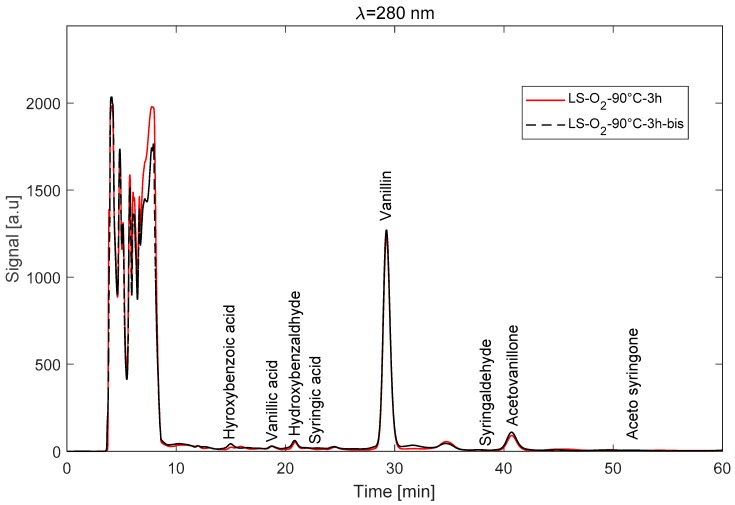
Example of a RP–HPLC chromatogram (90 °C–O_2_–3 h).

Before analyzing the effects of the different experimental parameters on the LS depolymerisation reaction, it would be interesting to check the reliability of the experimental procedure. Figure 2 shows the chromatograms of two repeatability tests done at 90 °C with oxygen for 3 h. The positions of the different monomers are indicated on the figure. The two signals are almost superimposed, denoting a good repeatability of the experiment and a good reliability of the protocol. The relative deviations between the two tests based on vanillin and acetovanillone concentrations were, respectively, 1.8% and 11.5%. The relative deviations between the two tests based on ${\bar{M}}_{w}$ and ${\bar{M}}_{n}$ were 7% and 11%, respectively. Also, the reactor was weighed before and after the experiment, and the relative weight difference was less than 5%, denoting the efficiency of the cooling system. Altogether, these repeatability results reflect the reliability of the experimental and analytical protocol. Those calculated deviations were taken as reference values for the estimation of the errors of the vanillin yield and average molecular weights.

Figure 2 also shows that vanillin is the main monomer produced during the depolymerisation reaction. The production of vanillin is accompanied by a production of acetovanillone and some *p*-hydroxybenzaldehyde. These identified molecules correspond to G and H units in the lignin. The absence of monomers related to the S unit in lignin is due to the nature of the parent wood, which is a softwood (with a high predominance of G units).

#### 2.2.1. Effect of Temperature

**Figure 3:**
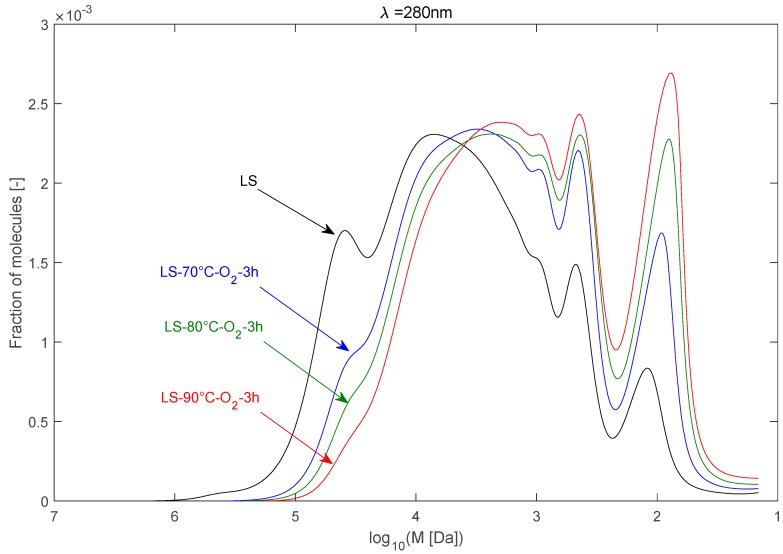
Molecular weight distribution of raw and depolymerized LS as a function of the reaction temperature (time = 3 h).

The effect of temperature on the depolymerisation of LS can be observed in Figure 3. It can be seen that the LS depolymerisation is occurring even at a temperature as low as 70 °C in an alkaline media under oxygen at atmospheric pressure. The SEC signal shows a clear decrease of the LS fragments having a molecular weight *M_w_* between nearly 10^3.5^ and 10^6^ Da, while the signal corresponding to the LS fragments with a *M_w_* < 10^4^ Da increased notably. The β-*O*-4 linkage is by far the most abundant linkage in native lignin. During sulfite pulping, this type of linkage is weakly degraded, which is depicted in the high *M_w_* of the raw LS. This type of bond is relatively weak and would be susceptible to breakage during the oxidative alkaline treatment, causing in part the depolymerisation of LS. Also, the depolymerisation can be due to other bond cleavage such as the α–β bond cleavage [22].

Increasing the temperature to 80 or 90 °C is accompanied by a greater LS depolymerisation. The peak between 10^3.5^ and 10^5^ Da is no longer visible at 90 °C. The signal increased visibly in the monomer region (*M_w_* around 150–200 Da), while it increased less-markedly between 10^2.5^ and 10^3.5^ Da, when increasing the temperature from 70 °C to 90 °C.

It is known from the literature that phenolic dimer structures such as β-1, β-5, β-*O*-4, α-carbonyl, β-*O*-4 and α-hydroxyl are reactive towards oxygen. These structures are known to exist in LS and can be classified by a reactivity order towards oxygen, with the β-*O*-4 structures being the most reactive [22]. In the case of lignosulfonates, there is an additional step involving the desulfonation reaction (involving SO_3_^−^ groups at C-α and C-γ positions). The removal of sulfonic groups leads to the formation of units with double bonds in propane lateral chain species that are naturally more reactive with O_2_ than the saturated counterpart [23].

Increasing the temperature will induce a higher reactivity of these structures and an enhanced depolymerisation. However, their non-phenolic homologues are much less reactive with oxygen, which implies that the extent of alkaline depolymerisation under oxygen depends on the parent structure of the lignin.

Altogether, one can see that the depolymerisation is occurring significantly in the range of 70–90 °C under an atmospheric pressure of oxygen. Considering the least-severe conditions, one can observe that the LS ${\bar{M}}_{w}$ dropped drastically from near 90,000 Da to about 30,000 Da after an alkaline oxidation at 70 °C with O_2_ for 3 h. This kind of oxidative treatment reduced the ${\bar{M}}_{w}$ by almost three-fold.

**Figure 4:**
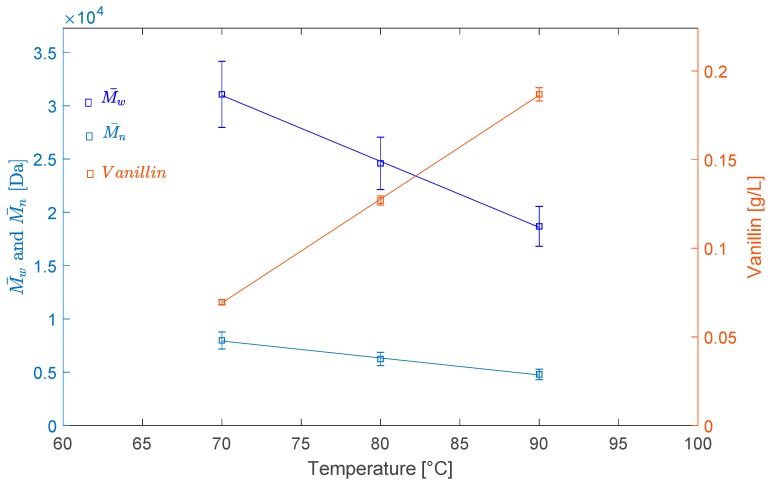
Effects of temperature on the LS average molecular weight and vanillin concentration in the solution.

The results also show that a 10 °C increase in the temperature results in a ${\bar{M}}_{w}$ decrease of about 6000 Da in the range of 70 °C to 90 °C. The average molecular weights decrease linearly with the temperature as depicted in Figure 4, and reach respectively 18,700 for ${\bar{M}}_{w}$ and 4800 for ${\bar{M}}_{n}$ Da at 90 °C.

In the same way, and as shown in Figure 4, the vanillin concentration increased linearly with the temperature from 0.07 g/L at 70 °C to 0.19 g/L at 90 °C. The mechanism of vanillin formation from lignin oxidation by oxygen is discussed elsewhere [24]. Similarly, the acetovanillone yield also increased linearly with the temperature (R^2^ > 0.99), but the concentration was much lower than that of vanillin, for which the selectivity was quite high. The formation of other low-molecular-weight non-identified compounds is quite probable, especially when there is a formation of phenolic radicals which react with oxygen leading to demethoxylation and aromatic ring-opening. Consequently, dicarboxylic acids such as muconic acid, muconolactone, maleic acid, succinic acid and malonic acid may be formed [25,26]. These molecules are not analyzed in the present work.

#### 2.2.2. Effect of Reaction Time

**Figure 5:**
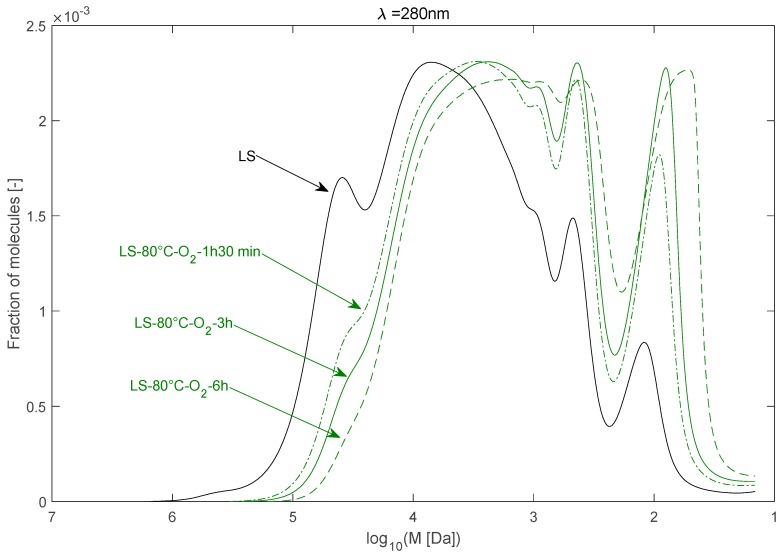
Molecular weight distribution of raw and depolymerized LS as a function of the reaction time (T = 80 °C).

The effect of time on the depolymerisation of LS can be observed in Figure 5. The temperature was maintained at 80 °C. The MWD of LS changed markedly since the first 1 h 30 min of reaction. The LS ${\bar{M}}_{w}$ dropped drastically from near 90,000 Da to about 30,000 Da after an alkaline oxidation at 80 °C with O_2_ for 1 h 30 min. This result, which is equivalent to what was obtained at 70 °C for 3 h, indicates that raising the temperature by 10 °C and halving the reaction time leads nearly to the same results.

The shift towards lower-molecular-weight LS fragments continues over time up to 6 h. The evolution is, however, less marked than that observed for the first 1 h 30 min of reaction. The peak related to low-molecular-weight molecules also became broader at 6 h of reaction.

**Figure 6:**
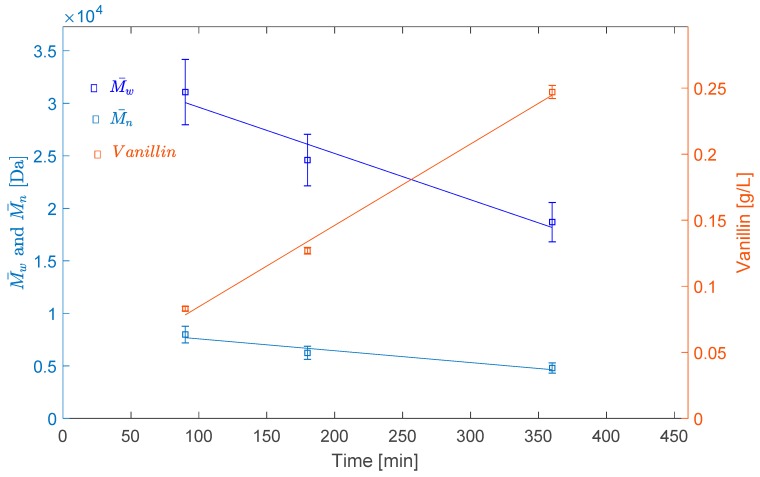
Effects of reaction time on the LS average molecular weight and vanillin concentration in the solutions.

In addition, the average molecular weights decreased nearly linearly with the reaction time within the tested range at 80 °C. ${\bar{M}}_{w}$ and ${\bar{M}}_{n}$ decreased, respectively, from 30,300 Da and 7800 Da to 17,200 Da and 4600 Da when increasing the reaction time from 1 h 30 min to 6 h (see Figure 6).

This linear decrease of the average molecular weight was accompanied by a linear increase of vanillin concentration. The concentration of vanillin increased from 0.08 to 0.25 g/L when increasing the reaction time from 1 h 30 min to 6 h (see Figure 6). It appears clearly that the decrease of the LS *M_w_* and the production of vanillin are correlated. The LS depolymerisation under O_2_ leads to the production of vanillin.

#### 2.2.3. Effect of H_2_O_2_ as Co-Oxidant

**Figure 7:**
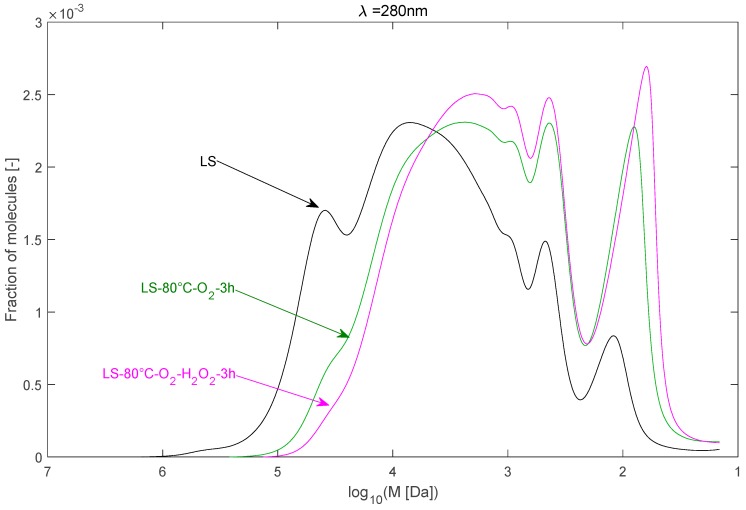
Effect of H_2_O_2_ addition on the molecular weight distribution of LS during the depolymerisation.

The effect of H_2_O_2_ as co-oxidant with O_2_ on the depolymerisation of LS is illustrated in Figure 7. The temperature was maintained at 80 °C. The MWD of LS after the alkaline depolymerisation under O_2_/H_2_O_2_ shifted slightly towards lower molecular weights. H_2_O_2_ appeared to enhance the depolymerisation of LS. The ${\bar{M}}_{w}$ decreased further to 16,000 Da compared to 24,600 Da under O_2_ only. However, the vanillin and acetovanillone concentrations remained nearly the same when adding H_2_O_2_. This may be due to different reaction pathways of H_2_O_2_ with LS which do not yield vanillin or acetovanillone, but probably other compounds. This would corroborate that the vanillin production route is mainly associated with O_2_.

The depolymerisation enhancement is probably due the action of HOO^−^ Indeed, in alkaline conditions, HOO^−^—which is the base form of H_2_O_2_ (pKa = 11.6 at 25 °C)—is the main active species. The high pH adopted in the present study (around 13) is justified to cause almost all of the H_2_O_2_ to take the form of HOO^−^.

HOO^−^ is a powerful nucleophile which selectively attacks electron-deficient carbonyl groups and conjugated carbonyl structures (aliphatic chains, mainly), in contrast with O_2_ or O_2_^●^^−^, which are electrophiles and preferentially attack electron-rich and olefinic structures by either a one- or two-electron transfer mechanism (aromatic cycle, mainly) [27]. This causes different reaction routes of H_2_O_2_ and O_2_ with LS.

It has been effectively shown that HOO^−^ is effective in lignin depolymerisation through a study on lignin model compounds. The proposed mechanism involves, at first, the oxidation of the alcohol group in the C-α position into a carbonyl group; the second step is the cleavage of the β-*O*-4 linkage by a series of nucleophilic attacks initiated by the reaction of the perhydroxyl anion on the carbonyl group [28].

Also, it has to be noted that H_2_O_2_ can undergo facile alkaline decomposition, which proceeds via a disproportionation reaction, leading to the formation of other active species (HO^−^, O_2_^●^^−^, HO^●^) that can react with LS.

This disproportionation leads to a rapid decomposition of hydrogen peroxide and the formation of a number of active oxygen species through an autocatalytic process. For instance, the superoxide anion radical, which is a powerful oxidant, can be formed via the reaction of a hydroxide radical and the perhydroxide anion.

#### 2.2.4. Effect of Cu^2+^ Catalyst in the Presence of O_2_

**Figure 8:**
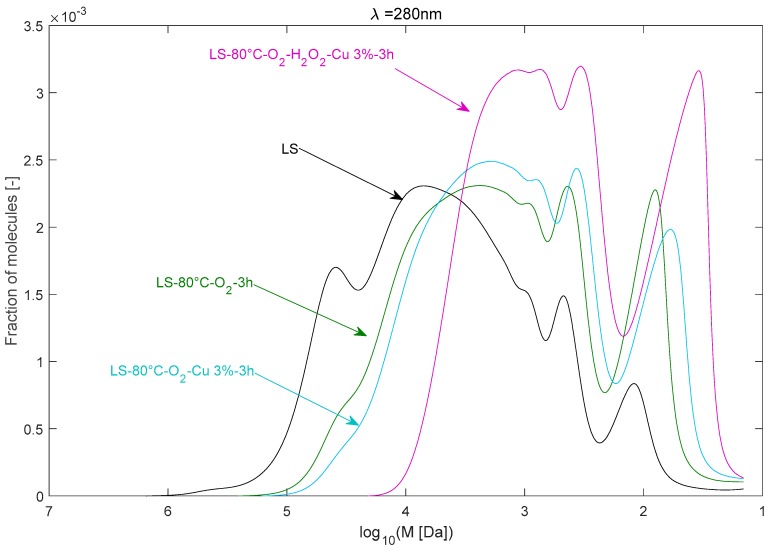
Effect of Cu^2+^ on the molecular weight distribution of LS during the depolymerisation under O_2_ and O_2_ + H_2_O_2_.

The effect of Cu^2+^ as catalyst in the presence of O_2_ on the depolymerisation of LS is illustrated in Figure 8. The temperature was maintained at 80 °C. The presence of Cu^2+^ affected the MWD of LS in the presence of O_2_. The signal showed a decrease of the intensity related to the higher-*M_w_* fragments, accompanied by an increase of the intensity related to the lower-*M_w_* LS fragments. The ${\bar{M}}_{w}$ decreased further to 17,300 Da compared to 24,600 Da under O_2_ only. Also, the vanillin concentration increased by almost three-fold when adding Cu^2+^ in the presence of oxygen. Cu^2+^ has been effectively reported to enhance the yield of phenolic aldehydes in the oxygen oxidation of lignins [25,29].

The effectiveness of Cu^2+^ as catalyst could be related to its high oxidation potential, which would promote electron-transfer processes from aromatic rings [14]. It can oxidize the phenolic structures that are recalcitrant to oxidation with O_2_. Cu^+^ can be then oxidized into Cu^2+^ by oxygen. The precise mechanisms by which Cu^2+^ can promote the lignin depolymerisation in the presence of O_2_ are not yet clear for us.

#### 2.2.5. Effect of Cu^2+^ Catalyst in the Presence of the O_2_/H_2_O_2_ System

The effect of Cu^2+^ as catalyst was much more remarkable in the presence of O_2_/H_2_O_2_. An outstanding change of the *M_w_* distribution of the LS after 3 h of reaction at 80 °C was observed in those conditions, as illustrated in Figure 8. Almost no LS fragments of a *M_w_* > 10^4^ Da subsisted after the treatment. The signal increased remarkably for *M_w_* < 10^3.5^ Da. High proportions of LS fragments of less than 5–6 aromatic units were obtained. The signal related to low-*M_w_* molecules also increased sharply and broadened in the same way.

The outstanding results obtained for the Cu^2+^/O_2_/H_2_O_2_ system are reflected in the values of ${\bar{M}}_{w}$ and ${\bar{M}}_{n}$, which dropped, respectively, from 90,000 and 16,000 Da to about 3500 and 1400 Da. The *PDI* decreased more than two-fold from 5.6 to 2.5. The vanillin concentration increased by two-fold compared to the case where only oxygen was used.

H_2_O_2_ is very sensitive to the presence of transition metals, which catalyze its decomposition into radicals. The formation of highly reactive radicals due to the Cu^2+^-induced decomposition of H_2_O_2_ is probably behind this higher depolymerisation extent. This explains the higher performance of the O_2_/Cu^2+^/H_2_O_2_ system compared to the O_2_/Cu^2+^ system. It was in fact shown that transition metals increased the reactivities of lignin model compounds with hydrogen peroxide in the order Mn^2+^ > Cu^2+^ > Fe^3+^, which is the same order of reactivity toward peroxide decomposition while Mg^2+^ stabilized the system [30]. Also, the authors found a wide variety of depolymerisation products obtained in catalyzed H_2_O_2_ depolymerisation of lignin model compounds. Vanillin and acetovanillone would probably represent a small part of the depolymerisation products. A GC–MS analysis would probably shed more light on the depolymerisation products.

It is also worthy to mention that part of the copper may also be complexed by the lignin itself, which would reduce the proportion of the decomposed H_2_O_2_ [31]. It is in fact known from the literature that Cu^2+^ can form strong complexes with lignin. For instance, it was demonstrated in [32] that the copper-binding capacity of a Kraft lignin at 25 °C was 87.05 mg/g. We may expect that on a lignin with some sulfonate groups (in the case of lignosulfonate), complex formation is even higher. This would stabilize the system further, wherein HOO^−^ is an active species in the depolymerisation reaction.

As a perspective, it could be interesting to study the addition of a bipyridine-type strong copper-complexing agent to minimize the decomposition of H_2_O_2_. This could minimize the proportion of decomposed H_2_O_2_ and reduce the amount of used copper.

#### 2.2.6. Summary of the Results

Altogether, these results demonstrate clearly the possibility of tailoring the molecular weight of LSs in rather soft conditions of temperature and oxygen pressure. The calculated average molecular weights of the initial and depolymerized LSs are summarized in Table 1.

We show here that it is possible, in rather soft conditions of temperature and oxygen pressure, to reduce the LS molecular weight by three to 25 times based on the initial LS ${\bar{M}}_{w}$. The PDI can then be decreased from 5.6 down to 2.5 in such a range. These results represent, to the authors’ best knowledge, a novelty in the field of lignin molecular-weight control, and open possibilities to obtain valuable oligomers from a high-molecular-weight LS that can be more suitable for use in applications such as adhesives, bioplastics, or others that require a reduction of the lignin molecular weight.

**Figure 9:**
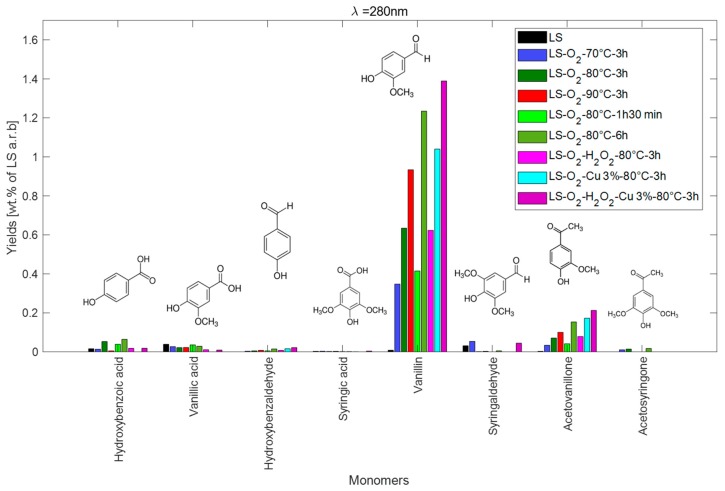
Yields of the different analyzed monomers (on as received basis a.r.b).

Concerning the monomers, Figure 9 shows the different analyzed monomer yields as a function of the experimental conditions. The selectivity to vanillin, measured as the ratio of the molar vanillin concentration to the molar concentration of all monomers, indicates that the vanillin production ranges between 73 and 88 mol % of the total identified monomers. This is followed, then, by acetovanillone, the production of which ranges between 7 and 13 mol % of the total identified monomers. The total yield of monomers varies between 0.5 and 1.70 wt % on a raw LS basis. The maximum yield was obtained when O_2_, H_2_O_2_ and Cu^2+^ were combined, which is in accordance with the highest depolymerisation extent. This may also open a novel route of vanillin production at atmospheric pressure, which may be optimized in terms of temperature, duration, and Cu^2+^ and H_2_O_2_ content.

## 3. Material and Methods

### 3.1. Lignosulfonates

Lignosulfonates were purchased from Roth in the form of 1000 g bottles. The moisture and ash content were estimated respectively at 6 wt % and 16.22 wt % according to the TAPPI (T550 and T 211) standard methods. XRF analysis showed that Na constitutes near to 96% of the minerals, with some traces of Ca, K, Cl, Si and Al. The chemical composition (weight % on as-received basis) is C (41.08%), H (5.23%), N (0.13%), S (4.87%), and O (32.47%), which was determined by difference to 100%.

### 3.2. Lab-Scale Experimental Facility for LS Deploymerisation

The lab-scale experimental facility comprises:

- The reactor (1) which is a 100 mL Duran bottle with a special spare screw cap for GL 45 stirred reactor. This cap has three upper entries: one along the revolution axis of the bottle and two inclined, others making a 45° angle with this axis.
- A magnetic stirrer with heating and ceramic heating plate (2) (IKA-C-MAG HS 7) to which is connected a contact thermometer ETS-D5 (3), enabling precise temperature control of the solution. This contact thermometer is introduced in the reactor via the vertical entry of the cap and is in direct contact with the solution. Hence, the temperature of the reacting medium is precisely controlled. The vertical entry is made hermetic ensuring no mass transfer through it.
- An oxygen supply system: The left inclined entry is connected to an O_2_ cylinder. The O_2_ flowrate is measured and controlled by a volumetric flowmeter (4) which is connected to this left entry via a special connector and flexible tubing. A small flexible tube is also introduced along this left entry. A standard pipette tip (50–1000 µL) is fixed to this tube, plunges into the solution and ensures the oxygen bubbling inside.
- A vapor condensation system (5): The right entry is connected to an Allihn condenser consisting of a long glass tube with a cool water jacket. A series of bulbs on the tube increases the surface area upon which the vapor constituents may condense, and hence ensures the reflux. An additional home-made electrical fan (6) is permanently blowing air on the lower part of the condenser to ameliorate the condensation.

### 3.3. LS Depolymerisation Experimental Conditions

In the present work, as we targeted to depolymerize LS in mild conditions of temperature (<100 °C) and atmospheric oxygen pressure, we chose to operate with a 2 M NaOH solution. This concentration is referred as a minimum for vanillin production via lignin alkaline oxidation using oxygen [23]. Higher soda molarity of 3 M was also adopted in [13], for instance.

To make the process of LS depolymerisation very attractive, we decided in the present study to investigate the reaction at lower temperatures (70, 80 and 90 °C), and at atmospheric pressure (no oxygen pressure). Knowing that the Kraft process operates at a minimum of 150 °C, the depolymerisation of LS by the caustic soda alone, if any, would remain marginal below 100 °C. This is why our reference tests are using caustic soda under oxygen atmosphere (no pressure) at temperatures as low as 70 °C.

To be successful in such mild conditions, and in particular to produce monomers of commercial interest (e.g., vanillin), it is necessary to reinforce the oxidizing power of the medium (production of vanillin and of most of the monomers detected in our study necessitates the use of strong oxidizing conditions). This is why we study also the effect of H_2_O_2_ as co-oxidant. The addition of H_2_O_2_ as co-oxidant of oxygen in pulp bleaching of sulfite pulp is well-documented [27]. The quantity ranges between 10% and 100% of the lignin to be treated. The H_2_O_2_/LS ratio in the present work is 56%.

Duration of oxygen bleaching experiment is usually close to 1 h in the industry. However, the reaction is performed at around 100 °C under oxygen pressure. Based on these practices, we chose in the present work longer durations from 1.5 to 6 h.

Finally, we know from the literature that the commercial production of vanillin from lignosulfonate requires high oxygen pressure, high temperature and the presence of copper which catalyzes the reaction and ameliorates the yield [12,13,14].

The effect of copper addition is also studied in our very mild conditions. We chose an excess of catalyst based on a 3 wt % Cu/LS ratio. The Cu was introduced as hexahydrated CuSO_4_.

The depolymerisation experiments are listed in Table 2. The effect of temperature between 70 and 90 °C is assessed in experiments 1 to 3, with O_2_ as oxidant and duration of 3 h. The effect of duration between 1.5 and 6 h is assessed in experiments 2, 4 and 5, with O_2_ as oxidant at a temperature of 80 °C. The effect of a co-oxidant, H_2_O_2_, is assessed in experiment 6, at 80 °C and for duration of 3 h. The effect of catalyst (Cu^2+^) is assessed in experiments 7 and 8, with O_2_ or O_2_/H_2_O_2_ as oxidant at 80 °C and 3 h of reaction time.

This set of experiments would bring preliminary results on the feasibility of the LS depolymerisation in mild conditions of temperature and oxygen pressure, as well as on the effect of Cu and H_2_O_2_ in LS depolymerisation in such conditions.

### 3.4. Unfolding of a Typical LS Depolymerisation Experiment

In all experiments, the mass of LS introduced in the reactor is near 1 g, weighed with an electronic scale with a precision of 0.1 mg. The total liquid volume is always 50 mL, giving thus a solid-to-liquid ratio of nearly 2%.

When oxygen alone is used, the entire liquid volume corresponds to a 2 M NaOH solution. The pH is around 13.6.

When H_2_O_2_ is used as co-oxidant with O_2_, a Sigma Aldrich aqueous 30% H_2_O_2_ solution is used. The weight ratio of H_2_O_2_/LS = 56 wt %. A micropipette is used to introduce the corresponding volume of the solution (near to 1.7 mL). The volume is afterward completed to the 50 mL mark using the 2 M NaOH solution.

When Cu is used as catalyst, a mass of hydrated CuSO_4_ (Sigma Aldrich, St. Louis, MO, USA), corresponding to a Cu/LS ratio of 3 wt %, is introduced in the reactor.

In a typical experiment, nearly 1 g of LS is weighed and introduced first in the reactor, then the hydrated CuSO_4_, or H_2_O_2_ (if used), then the 2 M NaOH solution to a final volume of 50 mL. The magnetic bar is introduced at the end. The reactor is then joined to the cap and closed, ensuring that the thermocouple and the pipette tip plunge inside the solution without touching the magnetic rod. The oxygen is then introduced with a flow rate of 0.33 mL/min in standard temperature and pressure conditions (STP).

The experiments are then launched for the defined duration after setting the final temperature of the solution (70, 80 or 90 °C). The characteristic time needed to reach 63% of the final temperature value is around 8–10 min and is taken into account in the heating program.

### 3.5. Monitoring the LS Depolymerisation: LS Molecular Weight Distribution and Monomer Concentration

One of the purposes of the present work is to develop analytical methods to quantify the depolymerisation extent of the LS. An HPLC analyzer (Thermo Separation Products, Waltham, MA, USA), equipped with a UV detector (UV 6000 LP), was used. Depending on the type of column, this analyzer can be used to perform size-exclusion chromatography (SEC) for the analysis of MWD of the LS (in an isocratic mode) or reverse-phase chromatography (RPC) to monitor the production of phenolic monomers after the depolymerisation. The two modes of analysis are discussed in the following sections.

#### 3.5.1. Operation in SEC Mode

SEC is a quite interesting technique for the characterization of lignins as it offers many advantages, such as wide availability, short analysis time, low sample demand, and determination of molar mass distribution of the lignin fragments over a wide range [33].

As we operate in an aqueous media for the depolymerisation experiments, the best option for an efficient SEC analysis was the aqueous-phase SEC. For an optimal separation of the LS fragments, 3 columns (Tosoh Bioscience, Tokyo, Japan: (1) TSKGEL G3000 PW; (2) TSKGEL G4000 PW; (3) TSKGEL G4000) were put in series. The columns (L × ID = 30 cm × 7.5 mm) are composed of spherical, hydrophilic polymethacrylate beads. The mean size of the beads in the TSKGEL G3000 PW is around 12 to 17 µm. The mean size of the beads in the TSKGEL G4000 PW is around 17 µm. The mean pore size is around 20 nm as given by the constructor. We also placed, before the first column, a guard column TSKGEL G2500PW-GMPW (L × ID = 7.5 cm × 7.5 mm, 13 μm particle size) to prevent degradation of the columns.

During the SEC analysis, ultrapure water at pH 12 (fixed by the addition of NaOH) was used as eluent. The flowrate was fixed at 0.6 mL/min and a 280 nm UV detection was used to get the signal. The analysis duration was 60 min. The signal was digitalized at a frequency of 1 Hz.

Ten polystyrene sulfonates with average molecular weight going from 970,000 Da down to 896 Da were used as calibrants; in addition, vanillin (152 Da) was added to the set of calibrants to locate monomer elution volume.

As we do not have commercial software for the SEC results treatment, we developed a homemade program in the MATLAB language. First, the program loads and reads the calibrant files, locates the maximum of the signal for each calibrant and searches for the corresponding elution volume. Then, it plots the calibration curve (${\bar{M}}_{w}$ vs. elution volume). A fourth-order polynomial describes it well (R^2^ > 0.99). The equation is determined by a nonlinear least-squares fitting method. Afterwards, the program loads and reads the files corresponding to the different LS depolymerisation experiments. The start and end of the elution curve are determined using a signal derivative method. A linear background subtraction is afterwards performed between these two limits. The calibration equation is then used to calculate the MWD of the lignin. The mean molecular weights in mass ${\bar{M}}_{w}$ and in number ${\bar{M}}_{n}$ are then calculated as follows:

$${\bar{M}}_{w}=\frac{\sum_{i=1}^{n}h_{i\;}M_{i}}{\sum_{i=1}^{n}h_{i\;}},$$

$${\bar{M}}_{n}=\frac{\sum_{i=1}^{n}h_{i\;}}{\sum_{i=1}^{n}\frac{h_{i\;}}{M_{i}}},$$

where $h_{i}$ and $M_{i}$ are, respectively, the signal value and molecular weight at the index “*i*”.

The polydispersity index is then given by:

$$PDI=\frac{{\bar{M}}_{w}}{{\bar{M}}_{n}}.$$

#### 3.5.2. Operation in RPC Mode

The RPC was used to monitor the potential monomer production during depolymerisation. We focused in the present work on eight main monomers that can be potentially produced from the oxidative alkaline depolymerisation of LS. These can be aromatic ketones (acetovanillone, acetosyringone), aldehydes (vanillin, syringaldehyde, *p*-hydroxybenzaldehyde) and acids (vanillic acid, syringic acid and *p*-hydroxybenzoic acid) corresponding to the G, S and H units of the lignin. [34]. These monomers were purchased in analytical-grade quality from Sigma Aldrich and used for the establishment of a method to separate and quantify them in RPC.

An Alltima C18-10U column (L × ID = 25 cm × 4.6 mm, particle size = 5 µm) was used for this purpose. The eluent was a mix of 90 vol % H_2_O (0.1% CH_3_COOOH) and 10 vol % CH_3_CN. The eluent flow rate was fixed at 0.6 mL/min and the column temperature was regulated at 50 °C. UV detection at 280 nm was used to identify and quantify these molecules.

Firstly, solutions of the different molecules were prepared and injected to identify their retention times. Checking that the resolution was good for the eight molecules, the method was validated and 4 concentration-level calibration curves were constructed for the concentration determination in LS depolymerisation solutions.

As for the SEC analysis, homemade software was developed in the MATLAB R2017a (MathWorks, Natick, MA, USA) language for the processing of the chromatographic data. The first part of the program is dedicated to the calibration curves. For each monomer, the program loads and reads the 280 nm UV signal obtained for the different calibration solution. Then, the different peaks are identified using a method based on the signal derivative to identify peak start and end. Afterwards, a linear background subtraction is performed between the two peak limits, and the integral area of the peak is calculated using the trapezoidal rule for the numerical peak integration. Calibration curves of area versus concentration are hence obtained. A linear dependence between these two variables was observed for all the monomers. The calibration equations were determined by linear least-squares fitting. The second part of the program is dedicated to the LS depolymerisation solutions, for which the program performs the same tasks and calculates additionally the corresponding unknown concentrations for each of the identified monomer peaks for which the elution time is known. Examples of calibration curves and of peak identification, background subtraction and area calculation are shown in Appendix A.

## 4. Conclusions

The present work demonstrated the possibility of controlling LS MWD via an alkaline oxidative depolymerisation at low temperatures and atmospheric pressure. Following the experimental conditions, a wide range of LS molecular weights can be obtained. The reduction of molecular weight was from three to 25 times based on the initial LS ${\bar{M}}_{w}$. The PDI can be decreased from 5.6 down to 2.5.

The depolymerisation reaction was discussed in light of SEC and RP–HPLC analyses, which were developed and validated in the present work. The results showed, for instance, that the LS average molecular weight dropped drastically from 90,000 Da to nearly 30,000 Da when treated at 70 °C under oxygen in a 2 M solution of NaOH. Raising the temperature or reaction time affected significantly the depolymerisation extent. Also, it was found that ${\bar{M}}_{w}$ and ${\bar{M}}_{n}$ were linearly dependent on temperature and time in the tested ranges, and so was the vanillin concentration. The reduction of LS molecular weight appears to be clearly accompanied by the production of vanillin.

Moreover, the addition of H_2_O_2_ as a co-oxidant or Cu^2+^ as catalyst caused a further decrease of the LS molecular weight. The combination of O_2_/Cu^2+^/H_2_O_2_ at 80 °C and for 3 h of reaction time induced the highest depolymerisation extent and phenolic monomer yield. With this kind of treatment ${\bar{M}}_{w}$ and ${\bar{M}}_{n}$ dropped, respectively, from 90,000 and 16,000 Da to about 3500 and 1400 Da. The PDI decreased more than two-fold from 5.6 to 2.5. The vanillin yield was about 1.4 wt % and the acetovanillone yield was about 0.21 wt % (LS raw basis). There was also a clear selectivity for the production of vanillin for all the tested conditions, which was found to be produced at relatively low temperatures. This relatively soft treatment of LS seems promising for tailoring the MWD for subsequent use in applications like adhesives and bioplastics. Also, the vanillin production in such relatively soft conditions may be a novel route to be further optimized.

## Figures and Tables

**Table 1:** Average molecular weights and PDI for the initial and depolymerized LS.

Sample ID	${\bar{M}}_{w}$ (Da)	${\bar{M}}_{n}$ (Da)	PDI (–)
LS	90,357	16,172	5.59
LS–70 °C–O_2_–3 h	31,072	7983	3.89
LS–80 °C–O_2_–3 h	24,595	6236	3.94
LS–90 °C–O_2_–3 h	18,688	4793	3.90
LS–80 °C–O_2_–1 h 30 min	30,336	7759	3.91
LS–80 °C–O_2_–6 h	17,148	4581	3.74
LS–80 °C–O_2_–H_2_O_2_–3 h	16,003	4479	3.57
LS–80 °C–O_2_–Cu 3%–3 h	17,286	4549	3.80
LS–80 °C–O_2_–H_2_O_2_–Cu 3%–3 h	3583	1425	2.51

**Table 2:** Experimental conditions for LS depolymerisation.

Experiment Number	Experiment Name	Temperature (°C)	Duration (h)	Oxidant	Catalyst Cu (wt % on LS Basis)
1	LS–70 °C–O_2_–3 h	70	3	O_2_	0
2	LS–80 °C–O_2_–3 h	80	3	O_2_	0
3	LS–90 °C–O_2_–3 h	90	3	O_2_	0
4	LS–80 °C–O_2_–1 h 30 min	80	1.5	O_2_	0
5	LS–80 °C–O_2_–6 h	80	6	O_2_	0
6	LS–80 °C–O_2_–H_2_O_2_–3 h	80	3	O_2_/H_2_O_2_	0
7	LS–80 °C–O_2_–Cu 3%–3 h	80	3	O_2_	3
8	LS–80 °C–O_2_–H_2_O_2_–Cu 3%–3 h	80	3	O_2_/H_2_O_2_	3

**Figure A1:**
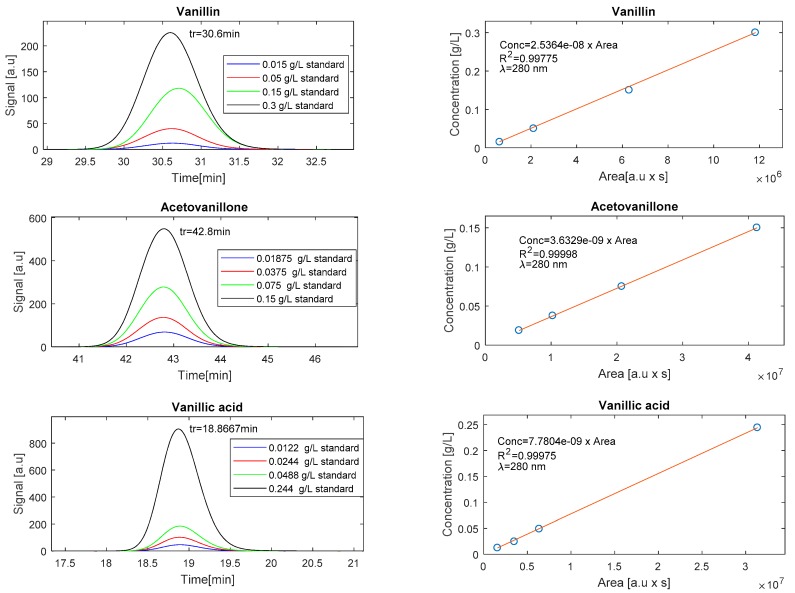
Calibration curves of some monomers.

**Figure A2:**
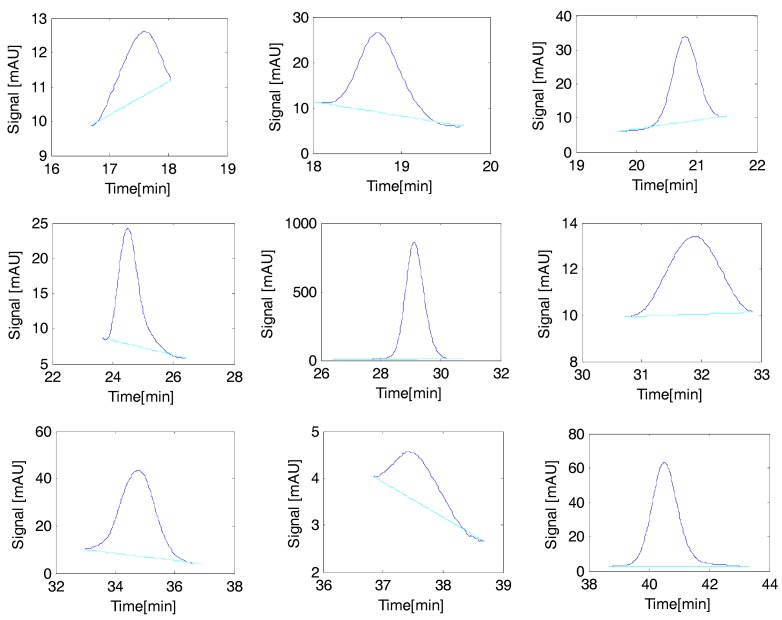
Example of peak identification and base-line determination for integration (peaks are in blue color and base lines are in cyan color).
